# The Physiological Response of Apricot Flowers to Low-Temperature Stress

**DOI:** 10.3390/plants13071002

**Published:** 2024-03-31

**Authors:** Jingjing Gao, Wenbo Guo, Qingwei Liu, Meige Liu, Chen Shang, Yuqin Song, Ruijie Hao, Liulin Li, Xinxin Feng

**Affiliations:** College of Horticulture, Shanxi Agricultural University, Taigu, Jinzhong 030801, China; gaojj@sxau.edu.cn (J.G.); gwbo0903@163.com (W.G.); 15582894301@163.com (Q.L.); liumeige265@126.com (M.L.); triciashang@163.com (C.S.); songyuqin_yyjc@163.com (Y.S.); hrj000@126.com (R.H.)

**Keywords:** apricot flowers, low-temperature stress, antioxidant enzymes, osmotic adjustment, cellular damage

## Abstract

The growth and development of apricot flower organs are severely impacted by spring frosts. To better understand this process, apricot flowers were exposed to temperatures ranging from 0 °C to −8 °C, including a control at 18 °C, in artificial incubators to mimic diverse low-temperature environments. We aimed to examine their physiological reactions to cold stress, with an emphasis on changes in phenotype, membrane stability, osmotic substance levels, and antioxidant enzyme performance. Results reveal that cold stress induces significant browning and cellular damage, with a sharp increase in browning rate and membrane permeability below −5 °C. Soluble sugars and proteins initially rise as osmoprotectants, but their content decreases at lower temperatures. Proline content consistently increases, suggesting a protective role. Antioxidant enzyme activities, including catalase (CAT), peroxidase (POD), superoxide dismutase (SOD), and ascorbate peroxidase (APX), exhibit a complex pattern, with initial increases followed by declines at more severe cold conditions. Correlation and principal component analyses highlight the interplay between these responses, indicating a multifaceted adaptation strategy. The findings contribute to the understanding of apricot cold tolerance and inform breeding efforts for improved crop resilience.

## 1. Introduction

The apricot flowers, a symbol of spring in China, are particularly vulnerable to low-temperature frost damage, especially in regions like Shanxi [1]. In recent years, sudden temperature drops during the blooming season have frequently occurred, hindering pollination and causing significant production losses [2]. To mitigate this threat, farmers have implemented strategies including improved orchard management and advanced weather tracking [3]. However, due to the uncertainty of climate change, late frost damage remains a significant issue in apricot production. Therefore, further research is needed to enhance the cold tolerance of apricot trees and ensure sustainable production.

Many studies have been dedicated to elucidating the mechanisms by which apricot flowers endure and respond to cold stress, as well as the genetic diversity in frost tolerance among various cultivars. In a study, Pakkish and Tabatabaienia [4] explored the application and mechanism of nitric oxide (NO) in preventing frost damage to apricot flowers. They demonstrated that NO treatment significantly reduced freezing injury, lipid peroxidation, and ion leakage, suggesting a role for NO in enhancing the antioxidant capacity of apricot flowers and altering solute content to improve cold tolerance. Kaya and Kose [5] utilized differential thermal analysis (DTA) to determine the cell death points (CDPs) in different apricot flower organs at subzero temperatures. Their findings revealed distinct sensitivities among various organs, with the receptacle being the most sensitive and the pistil the most tolerant, providing valuable insights into the structural vulnerabilities of apricot flowers to frost. Extending the research to a broader temporal scale, Kaya et al. [6] investigated the exothermic processes associated with late spring frost injury in apricot flower buds. Their work highlighted the changes in cold tolerance as the buds developed, indicating a dynamic response to environmental conditions. In a long-term study spanning 15 years, Szalay et al. [7] assessed the cold hardiness of apricot flower buds and flowers during the blooming period. They found that both genotype and year were significant factors affecting frost tolerance, with the later stages of flowering showing a greater influence of genotype. This research underscores the importance of considering both genetic and environmental factors in breeding programs aimed at improving frost resistance in apricot.

While previous studies have delved into the physiological reactions of apricot flowers to cold stress [8,9], a systematic and comprehensive understanding of the plant’s adaptive strategies remains elusive. There remains a gap in knowledge regarding the integrated response of apricot flowers to the full spectrum of low-temperature conditions, from mild to severe frost. A more thorough investigation is required to elucidate the complex interplay between various physiological responses and to identify the key factors that determine the success or failure of cold tolerance in apricot flowers.

This study aims to address this knowledge gap by conducting a comprehensive analysis of the physiological responses of apricot flowers to a gradient of low temperatures. The research will focus on several critical aspects, including changes in the phenotypic appearance and anatomical structure of the flowers, alterations in membrane permeability and lipid peroxidation, fluctuations in the content of osmoregulatory substances, and the enzymatic activities of the antioxidant system. By examining these elements in a systematic manner, we seek to uncover the underlying mechanisms that enable apricot flowers to withstand cold stress and to identify potential targets for genetic improvement or management practices aimed at enhancing cold tolerance.

## 2. Results

### 2.1. Changes in the Phenotype and Anatomical Structure of Apricot Flowers

**Figure 1:**
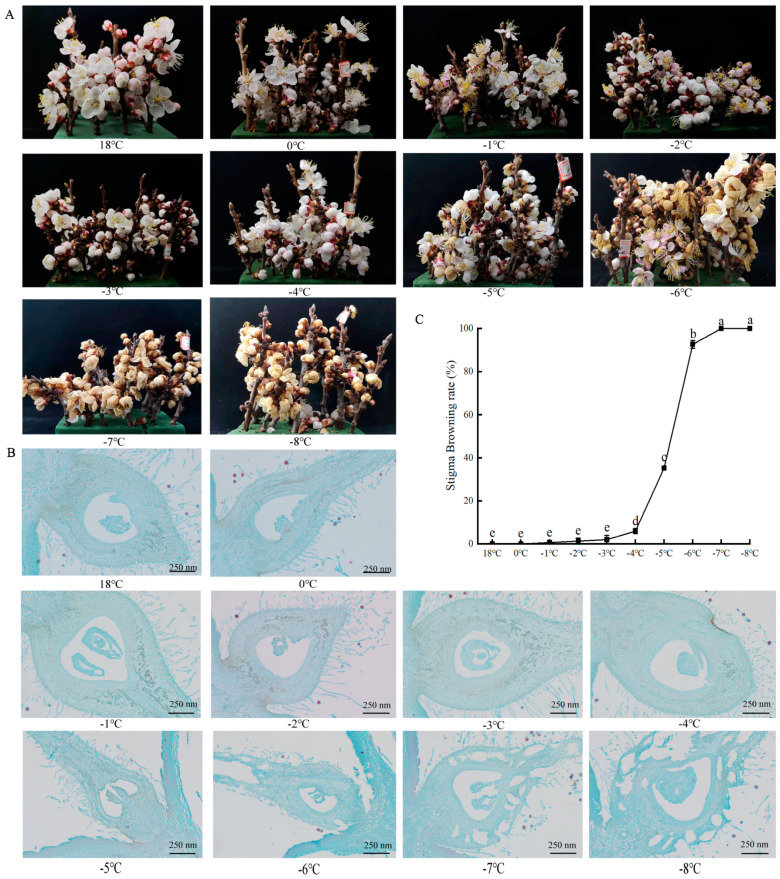
Browning phenomena in apricot flowers (**A**), anatomic structure of apricot ovaries (**B**), and browning rates analysis of apricot flowers (**C**) under low-temperature stress. Each data type has three sets of replicates, and superscripts of data in the different temperature treatments with the same lowercase letters indicate nonsignificant differences (*p* > 0.05).

The impact of low-temperature stress on the browning process of apricot flowers is depicted in Figure 1A. When subjected to temperatures ranging from 18 °C to −3 °C, the flowers exhibit minimal browning. However, as the temperature further descends to −4 °C to −6 °C, a noticeable increase in browning is observed. At temperatures between −7 °C and −8 °C, the flowers are almost entirely browned. Following exposure to frost, the apricot flowers initially take on a yellow-brown or black hue, with the scales becoming limp and loose. After a period of recovery at room temperature, they dry out and fall off. The petal tissue hardens upon initial freezing, and as the temperature warms, the petals gradually shift from white to yellow-brown before withering and dropping off. The pistils and stamens also undergo browning and drying, eventually curling up. At lower temperatures, the ovary may gradually wilt and shrivel due to freezing, leading to eventual detachment.

The influence of low-temperature stress on the anatomic structure of apricot flowers is illustrated in Figure 1B. Paraffin section observations of apricot flowers that have been subjected to low-temperature stress show that under mild low-temperature conditions (0 °C to −4 °C), there are no significant differences in the anatomical structure of the ovary compared to the control at 18 °C. However, at a treatment temperature of −5 °C, minor fissures or cavities start to form in the ovules and the ovary wall. As the temperature drops further (−7 °C to −8 °C), these fissures and cavities gradually increase, indicating a progressive worsening of damage to the apricot flowers.

Simultaneously, we assessed the browning rate of apricot flowers across a range of temperature conditions (Figure 1C). The rate remained relatively stable from 18 °C down to −3 °C, with no significant alterations observed. Yet, a marked escalation occurred as the temperature further descended; between −4 °C and −6 °C, the browning rate climbed significantly at a rate of 44.7%. This trend culminated in a complete browning of the apricot blossoms when subjected to temperatures between −7 °C and −8 °C, reaching a 100% browning rate.

### 2.2. Changes in Membrane Permeability and Membrane Lipid Peroxidation

**Figure 2:**
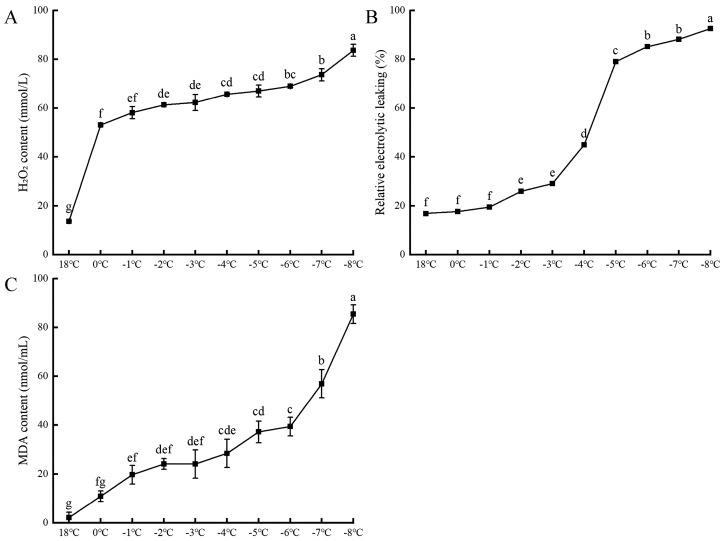
The effects of low-temperature stress on the hydrogen peroxide content (**A**), relative electrical conductivity (**B**), and malondialdehyde content (**C**) of apricot flowers. Each data type has three sets of replicates, and superscripts of data in the different temperature treatments with the same lowercase letters indicate nonsignificant differences (*p* > 0.05).

As shown in Figure 2, the relative electrical conductivity (REC) of apricot blossoms follows an “S”-shaped trajectory in response to the progressive drop in temperature. Within the range of 18 °C to −3 °C, there is a negligible rise in the relative conductivity. However, a marked increase is observed, with a 69.8% surge, as the temperature plummets from −4 °C to −6 °C, after which it plateaus at approximately 90%. In parallel, the accumulation of the metabolic byproduct hydrogen peroxide (H_2_O_2_) commences in earnest when the blossoms encounter low-temperature stress at 0 °C, escalating at a rate of 66%. Simultaneously, the levels of malondialdehyde (MDA), a marker of membrane lipid peroxidation, rise by 26.3% from 0 °C to −6 °C. This escalation is further compounded between −7 °C and −8 °C, with an accelerated rate of increase, reaching 71.2%. Consequently, the notable uptick in relative conductivity, H_2_O_2_, and MDA content with decreasing temperatures underscores the escalating vulnerability of apricot blossom cells to cold-induced damage.

The semi-lethal temperature (LT_50_), a critical threshold for plant survival, is determined by fitting a logistic regression model to REC data, which serves as a predictive indicator of a plant’s cold tolerance. In the context of this research, the semi-lethal low temperature for apricot blossoms in full bloom has been pinpointed at −5.2 °C, delineating the boundary of their frost resistance.

### 2.3. Changes in the Content of Osmoregulatory Substances

**Figure 3:**
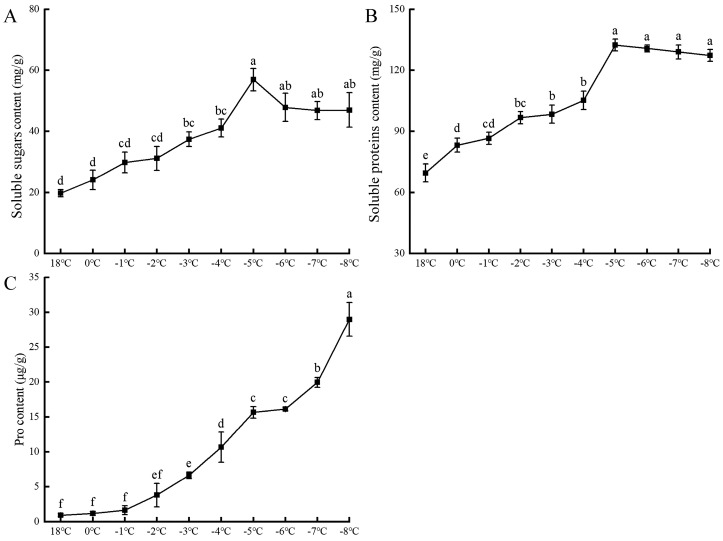
The effects of low-temperature stress on the soluble sugar content (**A**), soluble protein content (**B**), and proline content (**C**) of apricot flowers. Each data type has three sets of replicates, and superscripts of data in the different temperature treatments with the same lowercase letters indicate nonsignificant differences (*p* > 0.05).

Figure 3 illustrates that as the temperature progressively declines, the levels of soluble sugars (SSs) and soluble proteins (SPs) in apricot flowers exhibit an initial ascent followed by a descent. These components increase at rates of 34.3% and 96%, respectively, peaking at −5 °C with concentrations of 132.4 g/L and 56.9 mg/g. Beyond −5 °C, the continued temperature drop may impair the cells’ normal metabolic synthesis, resulting in a slight but nondramatic reduction in these contents. Conversely, the proline (Pro) content maintains an upward trajectory, escalating at a rate of 7.1% from 18 °C to −6 °C. This growth rate intensifies between −7 °C and −8 °C, with a 24.5% increase, potentially linked to disruptions in protein synthesis. Consequently, under low-temperature stress, apricot blossoms initially boost their SS and SP levels, which then peak and decline as temperatures fall further. In contrast, Pro content steadily ascends throughout the temperature range.

### 2.4. Changes in the Enzymatic Activity of the Antioxidant System

**Figure 4:**
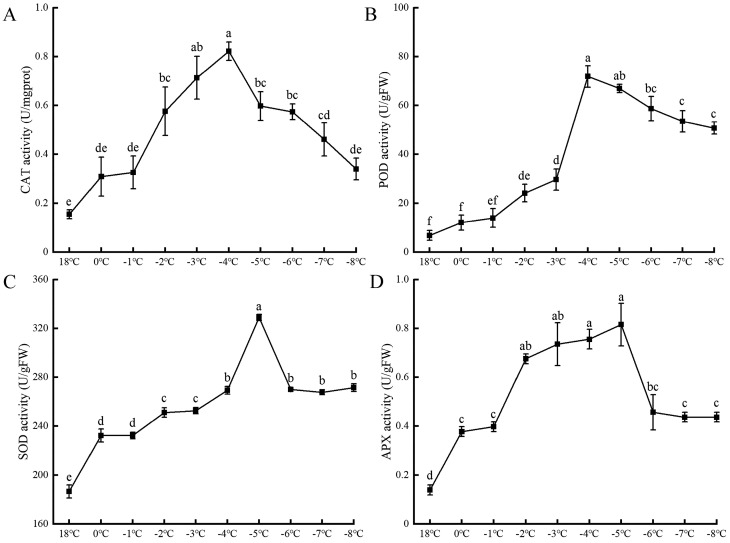
The effects of low-temperature stress on the catalase activity (**A**), peroxidase activity (**B**), superoxide dismutase activity (**C**), and ascorbate peroxidase activity (**D**) of apricot flowers. Each data type has three sets of replicates, and superscripts of data in the different temperature treatments with the same lowercase letters indicate nonsignificant differences (*p* > 0.05).

Figure 4 illustrates the impact of low-temperature stress on the activities of the antioxidant enzymes catalase (CAT), peroxidase (POD), superoxide dismutase (SOD), and ascorbate peroxidase (APX) in apricot flowers. As the temperature progressively drops, these enzymes’ activities undergo a dual response, initially spiking and then subsequently declining. CAT and POD activities climb at rates of 0.5% and 24%, respectively, from 18 °C down to −4 °C, where they reach peak values of 0.82 U·mgprot^−1^ and 71.9 U·g^−1^ FW, respectively. Beyond this point, their activities taper off at rates of 0.6% and 60.3%, respectively. In a similar vein, SOD and APX activities surge at rates of 250.6% and 0.5%, respectively, from 18 °C to −5 °C, achieving their maximum activities of 329 U·g^−1^ FW and 0.82 U·g^−1^ FW, respectively, at −5 °C. Following this peak, their activities diminish at rates of 284.7% and 0.5%, respectively. This suggests that CAT and POD may have distinct thresholds for low-temperature tolerance, peaking at −4 °C, while SOD and APX reach their optimal activity at −5 °C. Thus, within a specific low-temperature window, the antioxidant enzyme activities in apricot flowers initially ascend before exhibiting a decline.

### 2.5. Correlation Analysis

**Figure 5:**
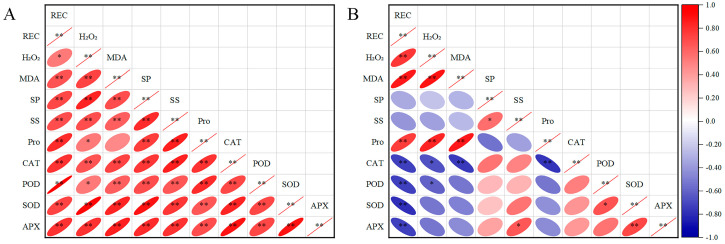
Correlation analysis of physiological indices of cold resistance in apricot flowers under 18 °C~−4 °C treatment (**A**) and −5 °C to −8 °C treatment (**B**). There are three replicates of each dataset and * in the graph indicates a significant correlation (*p* < 0.05) and ** indicates a highly significant correlation (*p* < 0.01).

Under low-temperature stress, the physiological indices of apricot flowers exhibit significant correlations that vary across different temperature ranges. As depicted in Figure 5A, within the temperature range of 18 °C to −4 °C, a strong positive correlation (*p* < 0.01) is observed between the osmotic adjustment substances in apricot flowers and the activity of the antioxidant enzyme system. The membrane permeability indicator REC is significantly positively correlated (*p* < 0.05) with the metabolic product H_2_O_2_ and shows an extremely strong positive correlation (*p* < 0.01) with other indicators, including the antioxidant enzyme POD and the membrane lipid peroxidation product MDA. Moreover, H_2_O_2_ is significantly positively correlated (*p* < 0.05) with the osmotic adjustment substance Pro, while MDA is extremely significantly positively correlated (*p* < 0.01) with all other indicators except Pro content.

In the more severe low-temperature stress of −5 °C to −8 °C (Figure 5B), REC of apricot flowers maintains an extremely significant positive correlation (*p* < 0.01) with H_2_O_2_, MDA content, and Pro content. However, it shows an extremely significant negative correlation (*p* < 0.01) with the activities of the antioxidant enzyme system, including CAT, POD, SOD, and APX. The positive correlation between H_2_O_2_ and both MDA and Pro content is more pronounced (*p* < 0.01), and there is a significant negative correlation with CAT and POD activities (*p* < 0.05). The positive correlation between MDA content and Pro content remains extremely significant (*p* < 0.01), while the negative correlation with CAT activity is equally pronounced (*p* < 0.01). There is a significant positive correlation (*p* < 0.05) between the contents of osmotic adjustment substances SPs and SSs, as well as between SS content and APX activity. Pro content is extremely significantly negatively correlated (*p* < 0.01) with CAT activity, while POD activity is significantly positively correlated (*p* < 0.05) with SOD activity. Additionally, a strong positive correlation (*p* < 0.01) is observed between SOD and APX activities. These findings shed light on the physiological adjustments that apricot flowers make to respond to low-temperature stress across varying cold conditions.

### 2.6. Principal Component Analysis (PCA)

PCA was performed on the physiological indicators of cold resistance in apricot flowers, yielding two principal components, each with an eigenvalue exceeding 1 (Table 1). These two principal components cumulatively account for 85.583% (>80%) of the variance in the physiological indicators of cold resistance, suggesting that they encapsulate 85.583% of the original data’s information without variable loss. The first principal component is defined by SP, SS, REC, H_2_O_2_, MDA, POD, and SOD, which together explain 67.962% of the original data’s information. The second principal component, determined by CAT and APX, represents 17.621% of the original data’s information. This analysis underscores the key factors contributing to the cold resistance of apricot flowers and their interrelationships.

**Figure 6:**
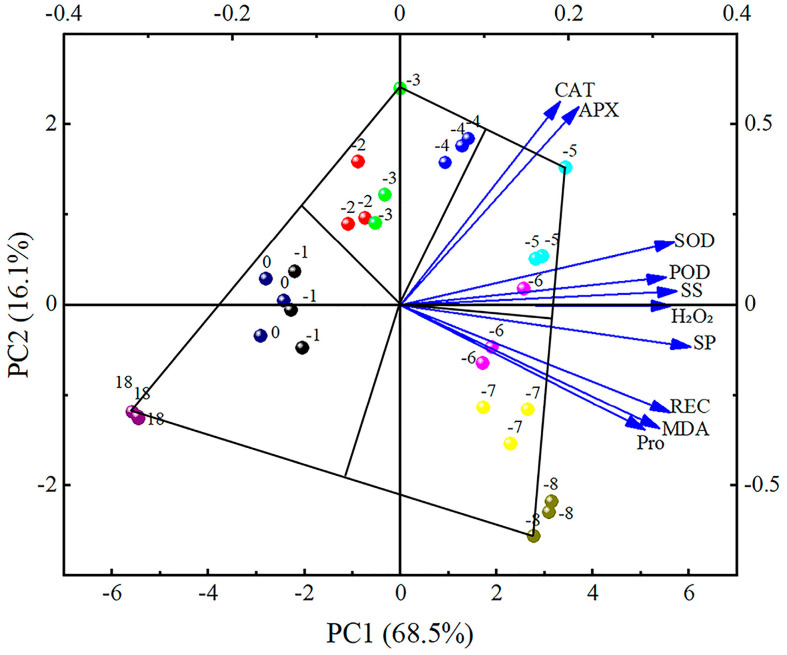
Interrelationship of physiological indicators in apricot flowers under various low-temperature stress conditions.

Based on the principal component analysis, a principal component double-labeled plot was made based on the first and second component values extracted from the information on the physiological indicators of apricot flowers. If the angle between different physiological indicators was less than 90°, it indicated a positive correlation, and if it was greater than 90°, it indicated a negative correlation. The results showed that there was a significant positive correlation between the physiological indicators of apricot flowers under low-temperature stress from 18 °C~−8 °C. The degree of frost damage in apricot flowers increased with decreasing temperature. By connecting the two treatments furthest from the origin in the same direction with a straight line, the final graph will form the closed polygon that will frame all the treatments in it. Then, making plumb lines from the origin to each side of the polygon, these plumb lines divide the whole double-labeled diagram into sectors. The four sectors show that at 18 °C~−4 °C, the cold resistance of the indicators is not outstanding; at −4 °C~−5 °C, the antioxidant enzyme systems CAT, POD, SOD, APX, and the osmotically conditioned substance SS is outstanding; at −6 °C~−8 °C, as the temperature continues to fall, the cell membrane is disrupted, membrane lipid peroxidation is aggravated, and the antioxidant enzymes are inactivated, resulting in the outstanding performance of SP, REC, MDA, and Pro indicators (Figure 6).

## 3. Discussion

The experimental results presented in this study offer a comprehensive understanding of the physiological responses of apricot flowers to low-temperature stress, revealing the intricate interplay between cellular damage, osmoregulation, and antioxidant defense mechanisms. The findings provide valuable insights into the adaptive strategies of apricot flowers and the critical thresholds for cold tolerance.

Numerous studies have demonstrated that the impact of low temperatures can lead to significant physiological and morphological changes in plants [8,10]. In this study, the phenotypic and anatomical changes observed in apricot flowers under low-temperature gradients are indicative of the severity of cold stress. The progressive browning and structural deterioration, particularly at temperatures below −5 °C, suggest a loss of cellular integrity and function. These changes are consistent with the increased browning rate and REC, which are markers of cellular damage. The accumulation of H_2_O_2_ and the rise in MDA content further confirm the oxidative stress and membrane lipid peroxidation experienced by the flowers, leading to cell death.

The increase in osmoregulatory substances such as soluble sugars and proteins up to −5 °C indicates an initial adaptive response to cold stress. These substances likely serve as osmoprotectants, helping to maintain cell turgor and stabilize cellular structures [11,12]. However, the subsequent decrease in content at lower temperatures may be due to the inhibition of normal metabolic processes, suggesting that the cold tolerance mechanisms of apricot flowers have limitations.

Proline (Pro), also a versatile osmoprotectant [13,14], is pivotal in enhancing plant resistance. It contributes to cellular osmotic equilibrium and structural integrity, particularly under stress [15,16]. Pro’s accumulation is instrumental in stabilizing proteins and membranes under various stress conditions, reducing oxidative stress [17,18]. Its antioxidant capabilities are crucial for neutralizing reactive oxygen species (ROS), thus protecting cells from oxidative harm [19,20]. These attributes render Pro an essential factor in plants’ adaptation to harsh conditions. In this study, the consistent increase in Pro content across all temperatures is particularly noteworthy. Its accumulation may be a key factor in the cold acclimation process of apricot flowers, although further research is needed to elucidate its exact role.

The enzymatic activities within the antioxidant system are instrumental in bolstering plant resilience [21,22]. Enzymes, including CAT, POD, SOD, and APX, synergistically combat the harmful effects of ROS, safeguarding plant cells from oxidative stress [23,24]. Their catalytic actions in the reduction and decomposition of ROS help to uphold the cellular redox equilibrium, which is essential for maintaining cellular integrity and enhancing the plant’s adaptive capacity to environmental challenges [25,26]. In the present study, the enzymatic activities of the antioxidant system showed a complex response pattern to low temperatures. The initial increase in activity up to −4 °C suggests an active defense against ROS generated during cold stress. However, the subsequent decline in activity at temperatures below −4 °C may indicate a saturation point or the inactivation of these enzymes due to extreme cold. The differential response of CAT and POD versus SOD and APX could be attributed to their distinct roles and sensitivities to temperature changes.

Correlation analysis revealed significant relationships between various physiological indicators, highlighting the interconnected nature of cold stress responses. The highly significant positive correlations between osmoregulatory substances and antioxidant enzyme activities, as well as between H_2_O_2_ and membrane permeability indicators, suggest a coordinated response to mitigate cold damage. The significant negative correlation between H_2_O_2_ and antioxidant enzyme activities, particularly CAT and POD, may indicate a regulatory mechanism where ROS levels are tightly controlled to prevent excessive oxidative stress.

PCA further supported the findings by identifying the key determinants of cold resistance in apricot flowers. The first principal component, which included SP, SS, H_2_O_2_, and MDA, reflects the overall cellular damage and osmotic stress. The second principal component, determined by CAT and APX, underscores the importance of antioxidant enzymes in the cold stress response. The PCA results indicate that while osmotic adjustment and antioxidant defense are crucial for cold tolerance, the effectiveness of these mechanisms is temperature-dependent.

This study focuses on the flowering stage because there is a significant difference in the tree’s sensitivity to cold stress between the preflowering period, when buds are dormant, and the peak blooming period. Typically, dormant buds, due to their reduced metabolic rate, possess enhanced cold resistance, allowing them to more effectively endure cold temperatures [6,7,8]. Furthermore, the capacity for cold resistance during the flowering period varies among different *Prunus* species. Certain species, such as the *Prunus sibirica*, demonstrate superior cold hardiness, attributed to their adaptation to chillier climates; in contrast, others like the *Prunus mume* show greater sensitivity to cold, likely due to their natural habitat in more temperate zones [27]. This diversity in cold resistance is presumably influenced by a combination of genetic predispositions and the distinct environmental challenges that have molded the evolutionary journey of each species.

In conclusion, the study provides a detailed analysis of the physiological responses of apricot flowers to low-temperature stress, highlighting the importance of temperature in determining the success of these responses. The findings suggest that while apricot flowers have certain adaptive mechanisms to cope with cold stress, there are critical thresholds beyond which these mechanisms are overwhelmed. This knowledge can be instrumental in developing strategies to improve the cold tolerance of apricot cultivars, potentially through breeding or genetic engineering approaches. Future research should focus on the molecular mechanisms underlying these responses and the identification of genetic markers for cold tolerance, which could lead to the development of more resilient apricot varieties for cultivation in colder climates.

## 4. Materials and Methods

### 4.1. Plant Materials

The apricots (*Prunus armeniaca* L. cv. Golden Sun) were sourced from the orchards of Taigu County, situated in Jinzhong City, Shanxi Province. The samples were harvested during early flowering stage when a flower’s buds have grown significantly and are on the verge of opening. To ensure methodological rigor, a selection criterion was established, mandating uniformity across orchard cultivation practices, tree vitality, environmental settings, and agricultural management standards, leading to the random selection of a cohort of 10 trees. From each of these trees, a set of 20–25 fruit-bearing branches, each measuring between 15 to 20 cm, were excised from the cardinal points—east, west, south, and north—at a standardized height of 1.5 m from the ground. After harvest, the branches were immersed in a 3% sucrose solution for an acclimatization treatment, with 50–60% of the apricot blossoms in full bloom. Subsequently, they were carefully transported to the laboratory for further experimental procedures.

### 4.2. Low-Temperature Treatment

Ten treatment temperatures were established at 18 °C, 0 °C, −1 °C, −2 °C, −3 °C, −4 °C, −5 °C, −6 °C, −7 °C, and −8 °C. Apricot short fruiting branches during the flowering stage were inserted into flower foam that had been soaked in water for 24 h (20 branches per block of foam, with each branch serving as one replicate). These were then placed in a PERCIVAL low-temperature incubator to simulate a low-temperature environment. The low-temperature incubator starts by cooling down first. Then, it slowly drops 1 °C every hour until it hits the target temperature, staying there for 1 h. After that, it warms up by 10 °C every 30 min until it reaches room temperature. Observations were made after a 12 h recovery period at room temperature. During the low-temperature stress, a small number of pistils were collected and fixed with FAA fixative. After a 3 h recovery at room temperature, a portion of the apricot flowers was used for the statistical analysis and measurement of browning rate and electrical conductivity (with the calyx removed). The other portion of the apricot flowers was preserved in liquid nitrogen and stored in a −80 °C ultra-low-temperature freezer for the determination of physiological indicators.

### 4.3. Measurement Methods

#### 4.3.1. Observation of Flower Phenotype and Browning Rate

After the low-temperature stress treatment, 100 flowers were randomly selected and observed for the degree of browning after a 3 h recovery period at room temperature. The browning rates of petals and stigmas were meticulously observed and documented. Floral organs that had suffered from frost damage took on shades of brown or black, in contrast to those that remained unscathed, which retained their fresh and vibrant green color. The results were statistically analyzed and the process was repeated three times.

#### 4.3.2. Observation of the Anatomical Structure of the Apricot Flower

The pistil materials, fixed with FAA (formaldehyde 38%, glacial acetic acid, and 70% alcohol in a ratio of 1:1:18), were dehydrated sequentially using ethanol of varying concentrations [28]. They were then cleared in a mixture of ethanol and n-butanol in different ratios, followed by infiltration with a solution of n-butanol and paraffin wax in varying proportions. Finally, the samples were embedded in embedding boxes, cooled, and subjected to continuous sectioning at a thickness of 8 μm. The sections were then stained with safranin and fast green and mounted.

#### 4.3.3. Relative Conductivity Determination

We weighed out 0.5 g of the treated fresh flowers, cut them into small pieces, and placde them into a conical flask containing 10 mL of distilled water. After allowing the mixture to stand for 24 h, it was shaken for 20 min to measure the initial electrical conductivity. Then, we immersed the flask in boiling water for 30 min. After cooling to room temperature, we measured the final electrical conductivity [29]. Measurement of electrical conductivity was performed by laboratory conductivity meter Cond 720 (WTW Inolab, Weilheim, Germany).

The calculation of the lethal temperature (LT_50_) was performed by measuring the relative electrical conductivity of apricot flowers after low-temperature stress [30]. The data were fitted to a logistic regression equation: Y = K/(1 + ae^−bx^). In this equation, Y represents the relative electrical conductivity, K is the asymptotic relative electrical conductivity, and a and b are parameters of the equation, with x denoting the temperature. By calculating the second derivative of the logistic regression equation and setting it to zero, the inflection point temperature (LT_50_) can be determined. The LT_50_ is then used to calculate the lethal temperature using the formula LT_50_ = (lna)/b.

#### 4.3.4. Membrane Lipid Peroxisome Determination

The determination of H_2_O_2_ and MDA in apricot flowers was conducted using assay kits from Nanjing Jiancheng, following the manufacturer’s instructions. Samples were ground in a mortar with liquid nitrogen, and 0.15 g was immediately weighed out after thorough grinding. This was then added to 10 mL of 0.1 mol/L phosphate buffer solution at pH = 7.8. After centrifugation at 4 °C, the supernatant was placed in an ice bath for the measurement of various physiological parameters. The reaction of H_2_O_2_ with molybdic acid produced a complex, and its absorbance was measured at 405 nm; MDA reacted with thiobarbituric acid to form a red product, and its absorbance was measured at 532 nm.

#### 4.3.5. Determination of the Content of Osmoregulatory Substances

Plant soluble sugars, soluble proteins, and proline were determined using the Plant Soluble Sugars, Soluble Proteins, and Proline Assay Kit (Nanjing Jiancheng, Nanjing, China), following the instructions. Samples were ground in a mortar with liquid nitrogen, and immediately after thorough grinding, 0.15 g was weighed out and mixed with 2 mL of 0.1 mol/L phosphate buffer solution at pH 7.8. After centrifugation at 4 °C, the supernatant was placed in an ice bath for the subsequent measurement of various physiological parameters. The -NH^3+^ groups of proteins were stained with Coomassie Brilliant Blue G-250, resulting in a purple color, and the absorbance was measured at 595 nm. Soluble sugars, under the action of concentrated sulfuric acid, reacted with anthrone to form blue–green sugar aldehyde derivatives, and their absorbance was measured at 630 nm. Proline reacted with acidic ninhydrin to produce a red product, and its absorbance was measured at 520 nm.

#### 4.3.6. Determination of the Activity of the Antioxidant Enzyme System

The plant CAT, POD, SOD, and APX activity assay kits (Nanjing Jiancheng, Nanjing, China) were used and the method was carried out according to the instructions. Samples were finely ground in a mortar with liquid nitrogen, and immediately after adequate grinding, 0.15 g was weighed out and combined with 2 mL of 0.1 mol/L phosphate buffer solution at pH 7.8. Following centrifugation at 4 °C, the supernatant was placed in an ice bath for the measurement of various physiological parameters. The reaction of CAT decomposing H_2_O_2_ was rapidly halted by the addition of ammonium molybdate, and the residual H_2_O_2_ reacted with the molybdate to form a pale-yellow complex, with absorbance measured at 405 nm. The activity of POD was determined using the guaiacol method, which is based on the principle of POD catalyzing the reaction of H_2_O_2_, with absorbance measured at 420 nm. SOD activity was assayed by the colorimetric method, where the superoxide anion O^2-^ is oxidized by the dye to form nitrite, resulting in a purple–red complex, and absorbance was measured at 550 nm. The activity of APX was measured by its catalysis of the reaction between ascorbic acid and H_2_O_2_, converting ascorbic acid to monodehydroascorbic acid, with absorbance measured at 290 nm.

### 4.4. Processing of Data

The test data were collated and calculated by WPS 2021, and one-way ANOVA, LSD, and Duncan multiple analysis comparisons and PCA were performed using SPSS 22 statistical software, with graphical and correlational analytical analyses completed with the aid of WPS 2021 and Origin 2021.

## 5. Conclusions

This research provided a detailed analysis of the physiological reactions of apricot flowers to low temperatures, highlighting the critical role of temperature in inducing cellular damage and the activation of defense mechanisms. The study observed significant browning, structural deterioration, and increased oxidative stress markers, such as H_2_O_2_ and MDA, indicating severe cold stress. The initial increase in osmoregulatory substances and antioxidant enzyme activities suggests an adaptive response, though this was limited by extreme temperatures. Correlation and principal component analyses revealed strong interconnections between these physiological indicators, pointing to a complex acclimation process. The findings are valuable for guiding future breeding efforts to develop cold-resistant apricot cultivars and for deepening our understanding of plant cold tolerance, which is crucial for agricultural sustainability in the face of climate change.

## Figures and Tables

**Table 1:** Principal component analysis results of apricot flower physiological indicators under various low-temperature stress conditions.

	PC1	PC2	PC3
Eigenvalue	8.36	2.17	0.56
Proportion %	67.96	17.62	4.57
Cumulative %	67.96	85.58	90.15

## Data Availability

Data are contained within the article.
